# Preclinical evaluation of sunitinib, a multi-tyrosine kinase inhibitor, as a radiosensitizer for human prostate cancer

**DOI:** 10.1186/1748-717X-7-154

**Published:** 2012-09-11

**Authors:** Colin Brooks, Tommy Sheu, Kathleen Bridges, Kathy Mason, Deborah Kuban, Paul Mathew, Raymond Meyn

**Affiliations:** 1 Department of Experimental Radiation Oncology, The University of Texas M. D. Anderson Cancer Center, Houston, TX, USA; 2 Department of Radiation Oncology, The University of Texas M. D. Anderson Cancer Center, Houston, TX, USA; 3 Department of Hematology-Oncology, Tufts Medical Center, Boston, MA, USA; 4 National University of Galway (NUIGalway), Galway, Ireland

**Keywords:** Sunitinib, Combinational therapy, Targeted therapy, Prostate cancer, Tyrosine kinase inhibitor, Radiation

## Abstract

**Background:**

Many prostate cancers demonstrate an increased expression of growth factor receptors such as vascular endothelial growth factor receptor (VEGFR) and platelet derived growth factor receptor (PDGFR) which have been correlated with increased resistance to radiotherapy and poor prognosis in other tumors. Therefore, response to radiation could potentially be improved by using inhibitors of these abnormally activated pathways. We have investigated the radiosensitizing effects of sunitinib, a potent, multi-tyrosine kinase inhibitor of the VEGFR and PDGFR receptors, on human prostate cancer cells.

**Methods:**

The radiosensitizing effects of sunitinib were assessed on human prostate cancer cell lines DU145, PC3 and LNCaP by clonogenic assay. Sunitinib’s ability to inhibit the activities of its key targets was determined by immunoblot analysis. The radiosensitizing effects of sunitinib *in vivo* were tested on human tumor xenografts growing in nude mice where response was assessed by tumor growth delay.

**Results:**

Clonogenic survival curve assays for both DU145 and PC3 cells showed that the surviving fraction at 2 Gy was reduced from 0.70 and 0.52 in controls to 0.44 and 0.38, respectively, by a 24 hr pretreatment with 100 nM sunitinib. LNCaP cells were not radiosensitized by sunitinib. Dose dependent decreases in VEGFR and PDGFR activation were also observed following sunitinib in both DU145 and PC3 cells. We assessed the ability of sunitinib to radiosensitize PC3 xenograft tumors growing in the hind limb of nude mice. Sunitinib given concurrently with radiation did not prolong tumor growth delay. However, when animals were treated with sunitinib commencing the day after fractionated radiation was complete, tumor growth delay was enhanced compared to radiation alone.

**Conclusions:**

We conclude, based on the *in vivo* results, that sunitinib and radiation do not interact directly to radiosensitize the PC3 tumor cells *in vivo* as they did *in vitro*. The fact that tumor growth delay was enhanced when sunitinib was given after radiotherapy was completed suggests that sunitinib may be acting on the irradiated tumor stroma and suppressing its ability to sustain regrowth of the irradiated tumor. Based on these preclinical findings, we suggest that the combination of sunitinib and radiation for the treatment of prostate cancer deserves further development.

## Background

External beam radiotherapy has been used to treat prostate cancers for more than five decades [1]; however, continued improvement in the use of this modality is warranted. The response of cancer cells to ionizing radiation may be modified by various strategies to improve antitumor effects. It is now understood that the expression of growth factor receptors such as vascular endothelial growth factor receptor (VEGFR) and platelet derived growth factor receptor (PDGF) may cause the increased resistance to the damaging effects of radiation [2]. VEGFR and PDGFR expression correlates with vessel density and poor prognosis in various tumors that exhibit resistance to cancer therapy. Although radiation enhances the expression of both VEGFR and PDGFR, combination studies using dual VEGFR/PDGFR inhibitors in conjunction with radiation, have demonstrated a marked enhancement of the antitumor effects [3,4]. Based on our expanding knowledge of signal transduction pathways in tumors, it is possible that the efficacy of radiotherapy could be improved by including agents that target VEGFR and PDGFR.

Sunitinib, a potent inhibitor of several tyrosine kinase receptors, has demonstrated both antitumor and anti-angiogenic activity. Preclinical biochemical and cellular assay studies tested its activity against different kinases and proved it to be a potent inhibitor of all three members of the VEGFR family, both PDGFR β and β, C-KIT, and Fms-like tyrosine kinase-3 (FLT3) [5].

Studies using human (H460, A431) derived xenograft tumors showed that a dose of 20–80 mg/kg/day of sunitinib resulted in tumor growth inhibition of 11-93% [6]. Human glioblastoma xenografts, treated with sunitinib at plasma concentrations of 50-100 ng/ml, exhibited a reduction in density and an increase in apoptosis in micro-vessels [7]. Inhibition of PDGFR phosphorylation and reduction in neovascularization have also been observed [8,9]. Previous reports also described sunitinib as an effective means to enhance the cytotoxic effects of ionizing radiation. Concurrent treatment attenuated the ERK and AKT pathways in pancreatic adenocarcinoma xenografts [10]. Furthermore, sunitinib reduced clonogenic survival in irradiated endothelial cells when compared to radiation alone. The synergy observed *in vitro* was confirmed under *in vivo* conditions using a hind limb xenografts tumor model, which resulted in a significant delay in tumor growth [11].

In the present study, this multi-tyrosine kinase inhibitor was tested on prostate cancer cells in order to evaluate its effectiveness at enhancing the antitumor effects of radiation. The results indicate that sunitinib enhances the radioresponse of human prostate cancer cells *in vitro* and *in vivo* but that the mechanism of this enhancement may be different in these two model systems.

## Methods

### Cell culture

The following three human prostate cell lines were obtained from American Type Culture Collection and evaluated for radiosensitization: PC3, DU145 and LNCaP. Both PC3 and DU145 cells were routinely maintained in RPMI 1640 medium while LNCaP cells were cultured in DMEM/F12 medium. All media was supplemented with 10% fetal bovine serum, 2 mM L-Glutamine and 100-units/ml penicillin-streptomycin, and all three lines were grown in an exponential growth phase at 37°C and 5% CO_2_ in a humidified atmosphere.

### Chemicals

Sunitinib was obtained from Pfizer Inc. in a powder form and aliquots were dissolved in DMSO and stored at −80°C.

### Western blot analysis

Cells were harvested two hours post-irradiation by trypsinization and centrifuged at 4°C for 10 minutes at 1100 rpm. Subsequent pellets were then resuspended in ice-cold lysis buffer (50 mM Hepes, pH 7.9, 0.4 mM NaCl, 1% NP-40, 1 mM EDTA, 2 μg/ml leupeptin, 2 M aprotinin, 5 M benzamidine and 0.5 mM/L phenylmethylsulfonyl fluoride). The protein concentration of each sample was determined by the DC protein assay (Bio-Rad laboratories, Hercules, LA), using a 96-well plate in a Perkin Elmer Wallac Victor 1420 plate reader. Equal amounts of protein were separated by 8-15% SDS–PAGE, transferred to polyvinylide difluoride membranes (Millipore, Billerica, MA) and blocked for 90 minutes in 5% nonfat dry milk TBS-T (0.05% v/v). Membranes were incubated overnight at 4°C in primary antibody including both total and phosphorylated forms of VEGFR, PDGFR, C-KIT, FLT3, AKT and ERK at a 1:1000 dilution in 5% BSA. Blots were washed three times and incubated with a horseradish peroxidase-conjugated secondary antibody (1:350 dilution; Amersham Biosciences) for 90 minutes. Blots were visualized by chemiluminescence with ECL-plus detection reagent (Amersham Biosciences, Arlington Heights, IL) according to manufacturer’s directions, on a Typhoon 9400 scanner (Amersham Biosciences, Piscataway, NJ).

### Clonogenic survival assay

Cells were seeded in T25 flasks and treated with sunitinib/DMSO at the indicated concentrations. Following various incubation periods, non-confluent cultures of LNCaP, PC3 and DU145 cells were irradiated using a ^137^Cs source. Cells were trypsinized, counted, and known numbers were re-plated in 60 mm dishes in triplicate and returned to the incubator. For DU145 and PC3, colonies were stained with crystal violet 12 days later with a longer incubation of 18 days allowed for LNCaP cells. Colonies consisting of 40 or more cells were counted and the percentage plating efficiency and surviving fraction for a given radiation dose were calculated based on the survival of non-irradiated cells treated with either drug or vehicle alone.

### Mice

Male nude (nu/nu) mice were used for the *in vivo* studies. They were bred in the Experimental Radiation Oncology specific-pathogen free barrier mouse facility and were 3–4 months of age at the start of the experiments. Mice were housed 3–5 per cage, exposed to 12-hour light dark cycles, and given free access to sterilized pelleted food (Prolab Animal Diet, Purina Mills Inc., St. Louis, MO) and sterilized water. Animals were maintained in an Association for Assessment and Accreditation of Laboratory Animal Care approved facility, and in accordance with current regulations of the United States Department of Agriculture and Department of Health and Human Services. The experimental protocol was approved by, and in accordance with, institutional guidelines established by the Institutional Animal Care and Use Committee.

### Tumor implantation and antitumor efficacy studies

Solitary tumors were produced by inoculation of 1 x 10^6^ PC3 cells into the right hind leg of mice. When tumors grew to 7 mm in diameter (range 6.8-7.3 mm), mice were randomized into groups and treatment initiated as follows: 1) vehicle only, 2) sunitinib only, 3) local tumor irradiation (XRT), 4) a combination of sunitinib and XRT or 5) no treatment (control). Groups consisted of 4 to 8 animals each. Sunitinib was given at a dose of 1.2 or 1.3 mg/mouse daily for 5 days by oral gavage using 2 different protocols: either 1 h before each dose of radiation or starting 24 h following the last dose of radiation. Radiation was delivered in 5 daily fractions of 1 or 3 Gy. Tumor bearing mice were locally irradiated without anesthesia using a small animal irradiator (Atomic Energy of Canada, Ottawa, Canada) consisting of parallel opposed ^137^ Cs sources, at a dose rate of 5 Gy/min.

Tumor growth delay was the endpoint used to determine antitumor efficacy of the treatments. To obtain tumor growth curves, three mutually orthogonal diameters of tumors were measured 2–3 times/week with a vernier caliper, and the mean values were calculated. Tumor growth delay plots were generated depicting average tumor diameter as a function of days after initial treatment. Tumor bearing mice were euthanized by CO_2_ inhalation when tumors grew to 14–15 mm diameter. Regression and subsequent regrowth of tumors was expressed as the time in days for tumors in the treated groups to grow from 7 mm to 12 mm in diameter minus the time in days for tumors in the control group to reach the same size. This was termed absolute growth delay (AGD).

### Immunofluorescence staining

For detection of radiation-induced DNA double strand breaks (DSBs) by γH2AX foci, we used a procedure reported previously [12]. Briefly, cells were grown overnight on cover slips in 35 mm dishes and treated for varying time periods in sunitinib. Dishes were irradiated with 2 Gy using a ^137^Cs source. At varying time points, medium was aspirated and cells were washed in PBS (Ca and Mg free) for 5 minutes. Cells were then fixed with 1% paraformaldehyde for 10 minutes followed by submersion in 70% ethanol for another 10 minutes. Following fixation, cells were incubated in 0.1% NP-40 (made in PBS) for 20 minutes before two five-minute washes and placed in 5% BSA (in PBS) blocking buffer for 30 minutes. Following blocking, primary antibody for γH2AX was prepared in 5% BSA-PBS at a 1:300 dilution. Incubation lasted 2 hours with gentle shaking at room temperature. Cells were subsequently washed four times at 10 minutes each in PBS before incubation for 30 minutes in FITC- labeled secondary antibody at a dilution of 1:300 (γH2AX) in 5% BSA-PBS. Incubation was followed by another four washes at 10 minutes each in PBS. Cells were subjected to DAPI (44’, 6-diamidino-2-phenylindole 1 ug/ml) in PBS for 5 minutes. Following the fourth and final wash cover slips were removed from the dishes and placed onto antifade solution mounted slides. Slides were sealed and examined using a Leica fluorescence microscope. The number of foci was manually counted in at least 40 cells per sample. Each independent experiment was repeated 3 times.

### Statistical analysis

The averages of at least three independent experiments were used in each independent study. Data was analyzed using the paired *t*-test and described as +/− standard error (SE). A difference of p<0.05 was deemed as significant.

## Results

### RTK expression in three prostate cell lines

**Figure 1:**
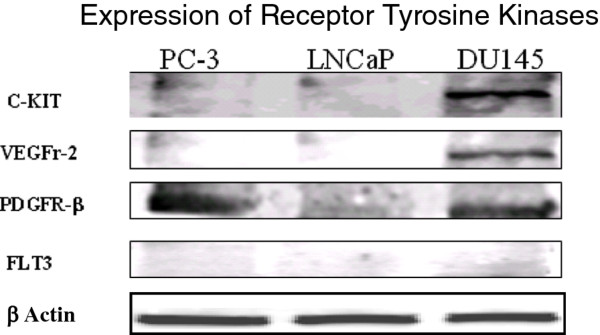
**Expression of RTK’s detected by western blot analysis.** Three prostate cell lines PC3, LNCaPs and DU145 were assessed for the presence of RTKs, shown to be targets of sunitinib (c-KIT, VEGFR-2, PDGFR- and FLT3). DU145 was found to be positive for all except for FLT3. PDGFR- was the only RTK found to be expressed in PC3 cells while LNCaPs were found to be negative for all four.

As stated, sunitinib has been shown to be a potent inhibitor of certain receptor tyrosine kinases (RTK’s) including VEGFR2, PDGFR-β, c-KIT and FLT3. We determined the expression levels of these receptors in all three prostate cell lines by western blot analyses (Figure 1). DU145 cells were found to be positive for VEGFR2, PDGFR- and C-KIT. PC3 cells were found to be only positive for PDGFR-, while LNCaPs proved to be negative for all four receptors. FLT3 was not expressed by any of the three cell lines.

### Inhibition of its cellular targets using sunitinib

**Figure 2:**
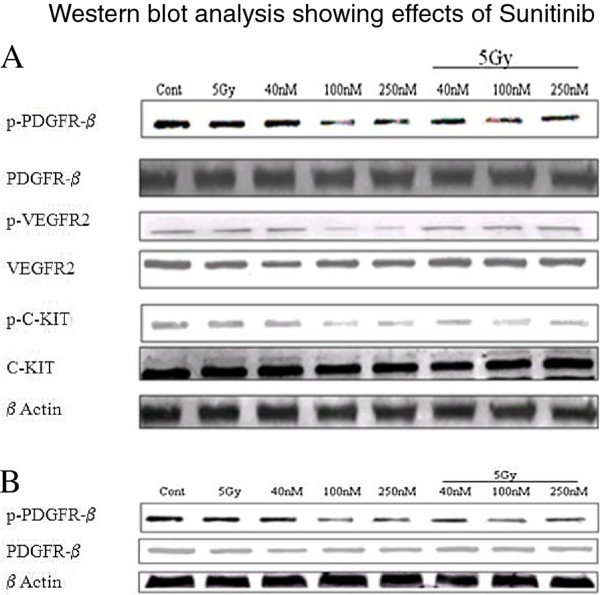
**Western blot analysis of the effects of sunitinib, with or without irradiation, on three of its trans-membrane receptor targets.** Both total and phosphorylated forms of PDGFR-β, VEGFR2, and C-KIT were detected by western blot analysis. Both DU145 (**A**) and PC3 (**B**) cells were treated with sunitinib (40, 100, 250 nM for 24 hours) and harvested for western blot analysis 2 h after irradiation. β-Actin was used as a loading control.

We next tested whether sunitinib inhibited the activation of these targets in the cell lines under investigation. Decreased levels of p-PDGFR-β, p-VEGFR2 andp-C-KIT were observed in un-irradiated DU145 cells following a 24 hour pretreatment with both 100 and 250 nM sunitinib (Figure 2A). Decreased levels of p-PDGFR-β were also observed in un-irradiated PC3 cells following a 24-hr pretreatment with both 100 and 250 nM sunitinib (Figure 2B). In irradiated DU145 samples, 100 nM sunitinib reduced the phosphorylation of both p-C-KIT and p-PDGFR-β, below the level of both control and radiation alone. Sunitinib although effective at reducing the expression of p-VEGFR2 at a concentration of both 100 and 250 nM, did not appear to reduce the expression when combined with 5 Gy (Figure 2A). Both 100 and 250 nM of sunitinib in combination with 5 Gy was found to be effective at reducing the expression of p-PDGFR-β when compared to control and radiation alone in the PC3 cell line (Figure 2B).

### Radiosensitization determined by clonogenic survival assays

**Figure 3:**
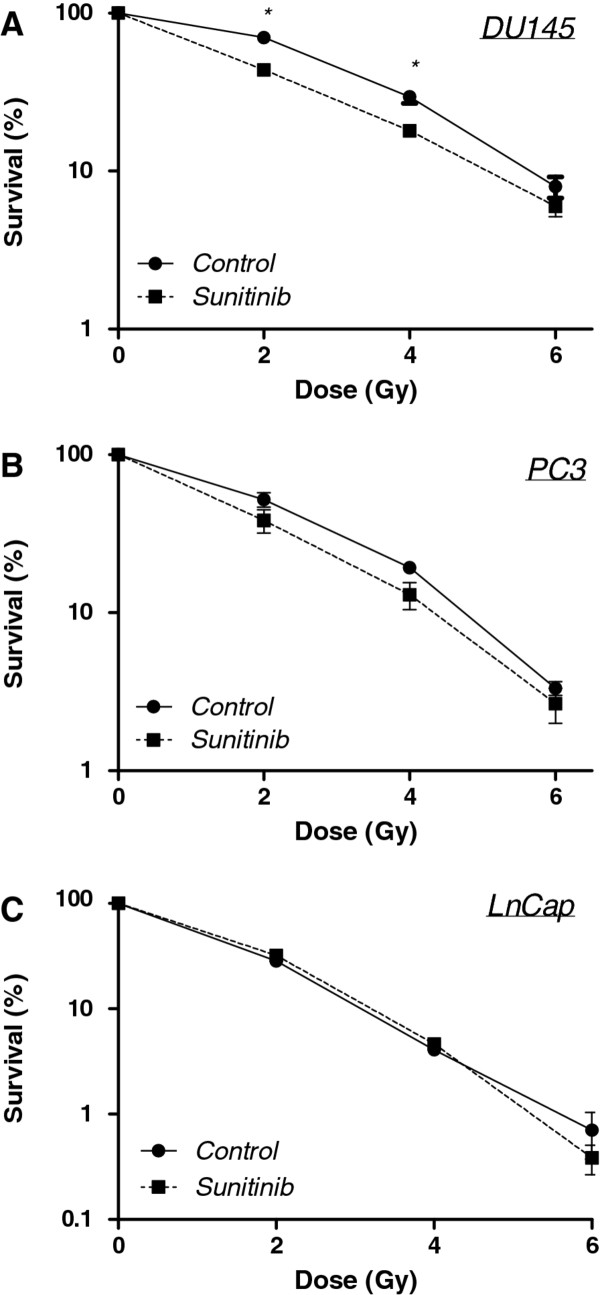
**Assessment of radiosensitization by sunitinib using clonogenic survival assays.** Sunitinib radiosensitized both DU145 (**A**) and PC3 (**B**) cells using concentrations of 100 nM for the DU145 cells and 250 nM for the PC3 cells. LnCap (**C**) cells were not radiosensitized with 250 nM. Cells were treated with sunitinib for 24 hrs and assessed for radiosensitization by clonogenic cell survival immediately after irradiation. Each data point represents the average of three independent experiments each plated in triplicate. * indicates *p* < 0.05.

We assessed the radiation enhancing effects of sunitinib by use of clonogenic survival assays. For the DU145 cells, following a 24-hour incubation period, the survival fraction at 2 Gy (SF2) was reduced from 0.70 in the control cells to 0.44 in 100 nM sunitinib-treated cells (Figure 3A). The radiosensitizing effect of sunitinib on DU145 cells was not further increased by using doses higher than 100 nM drug (data not shown). For PC3 cells (Figure 3B), the optimum dose range was found to be between 100 nM and 250 nM; doses higher than 250 nM had no further radiosensitizing effects. Using a 24-hour pretreatment with 250 nM of sunitinib the SF2 was reduced from 0.52 in the control to 0.38 in the treated sample. Only a slight but insignificant difference was observed in respect to varying incubation periods for the sunitinib treatments (data not shown). Sunitinib did not exhibit a radiosensitizing effect on the LNCaP cell line (Figure 3C), correlating with the lack of targets in these cells as was shown in Figure 1. In addition to calculating SF2 values, we also calculated the dose enhancement factors (DEF), that is, the ratio of doses required to reduce survival to 10%. The DEF values for DU145 and PC3 were both 1.1 whereas the DEF value for the LnCaP cells was 1.0. At the nM concentrations used in these experiments, sunitinib alone did not lower the plating efficiencies for the cell lines examined.

### Inhibition of downstream signaling

**Figure 4:**
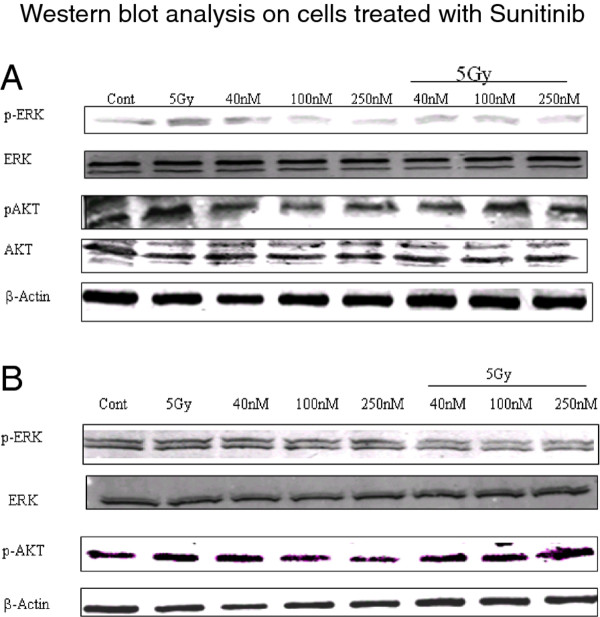
**The effects of sunitinib on downstream signals.** Both DU145 (**A**) and PC3 (**B**) cells were treated with sunitinib (40, 100, 250 nM) and harvested for western blot analysis 2 hours after irradiation (5 Gy). Cells were assessed for the effect of sunitinib on two key signaling proteins, ERK and AKT; both of which are involved in the radiation response and lie downstream of all three RTK targets, p-PDGFR-β, p-VEGFR2, and p-C-KIT. Actin was used as a loading control.

Radiation-induced phosphorylation of both ERK and AKT was observed in DU145 cells (Figure 4A) but not in PC3 cells (Figure 4B). With respect to p-ERK, sunitinib, at all 3 concentrations tested, suppressed p-ERK activation in the sunitinib + radiation samples compared to the 5 Gy only samples in both cell lines. With respect to p-AKT, expression was reduced in the sunitinib treated samples for the DU145 cells but this suppression was not maintained in the sunitinib + radiation samples.

### Immunofluorescence staining for γH2AX foci

**Figure 5:**
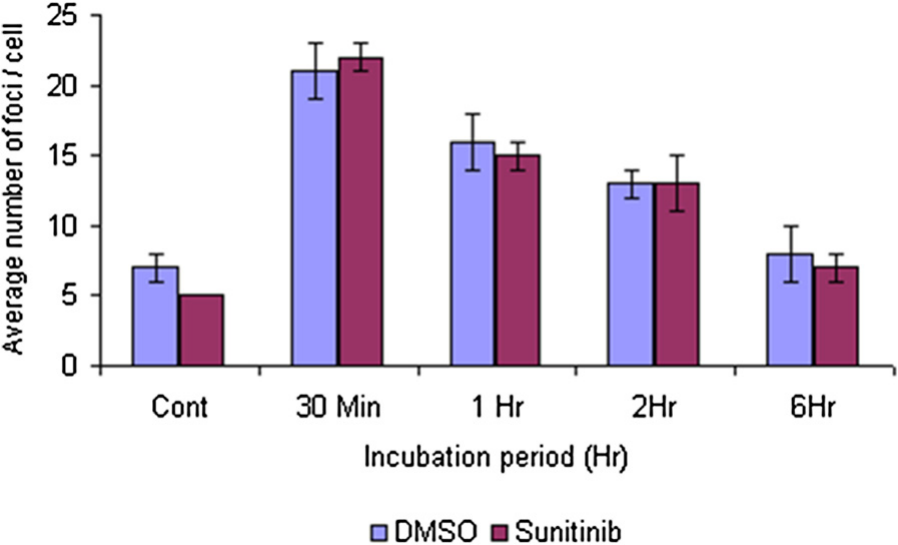
**Immunofluorescence detection of γH2AX stained DNA repair foci.** DU145 cells were treated for 24 hrs with 100 nM sunitinib and irradiated with 2 Gy. Cells were harvested at the indicated times and stained for γH2AX. Foci per cell were counted in 40 cells per sample. Error bars represent standard error.

Cells were harvested at given time points post radiation in order to detect if sunitinib resulted in the persistence of radiation-induced DNA double strand breaks (DSBs) detected on the basis of γH2AX foci. Radiation-induced γH2AX foci were detected in DU145 cells 30 min following 2 Gy irradiation (Figure 5) and the level of foci decreased with time over the next 6 hrs indicating repair of the DSBs. Sunitinib treatment, however, did not alter either the induction or subsequent disappearance of foci at any time examined suggesting that sunitinib does not affect the repair of radiation-induced DSBs. An identical experiment was conducted using PC3 cells and, similar to the case for DU145 cells, sunitinib did not alter the kinetics of γH2AX foci induction or disappearance in these cells either (data not shown).

### *In vivo* studies

**Figure 6:**
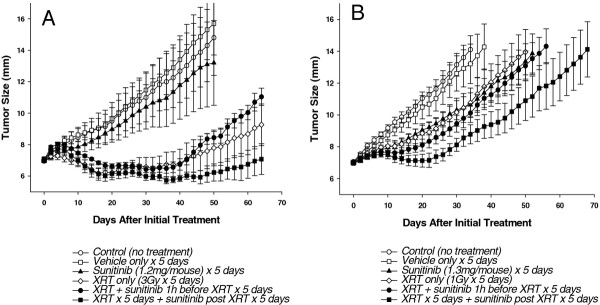
**Tumor growth delay curves for PC3 xenografts treated with the combination of sunitinib and radiation.** Tumors were generated by inoculation of PC3 cells into legs of nude mice and treatments initiated when tumors grew to 7 mm. Panel **A**: untreated control, vehicle only for 5 days, sunitinib (1.2 mg/mouse) only for 5 days, local tumor radiation only (3 Gy daily for 5 days), sunitinib for 5 days 1 h before each radiation dose, sunitinib for 5 days beginning 1 day after completion of 5 radiation doses. Panel **B**: untreated control, vehicle only for 5 days, sunitinib (1.3 mg/mouse) only for 5 days, local tumor radiation only (1 Gy daily for 5 days), sunitinib for 5 days 1 h before each of 5 radiation doses, sunitinib for 5 days beginning 1 day after completion of 5 radiation doses. Each data point represents average tumor diameter for 6–8 mice; bars, SE.

We assessed the ability of sunitinib to radiosensitize PC3 xenograft tumors growing in the hind limb of nude mice. Radiation doses were delivered to 7 mm-diameter tumors in 5 daily fractions of 1 or 3 Gy. In the first set of experiments, sunitinib was given by gavage as 1.2 mg/mouse for 5 days concurrent with fractionated irradiation or following the completion of radiation. The animals were followed for several weeks after treatment and tumor growth curves were generated for the different treatment groups (Figure 6A). The results show that sunitinib by itself produced a slight but not statistically significant growth delay compared to untreated controls. Fifty days after initial treatment, the average tumor size was 14.8 mm (±2.4) for untreated controls, 15.7 mm (±1.5) for mice treated with vehicle alone and 13.1 mm (±2.7) for mice treated with sunitinib alone. Radiation, by itself, produced significant tumor growth delay; average tumor size on day 50 was only 7.8 mm (±1.1) (*p* = 0.01). Mice with tumors that received irradiation were followed for 64 days following initiation of treatment. Tumors in irradiated mice at 64 days were 9.3 mm (±1.3) after radiation only, 11.0 mm (±0.5) after sunitinib given concurrent with radiotherapy and 7.1 mm (±1.0) when sunitinib was delivered post radiation treatment. Thus, sunitinib given concurrently with radiation did not prolong tumor growth delay, while sunitinib treatment initiated after the completion of fractionated radiation appeared to enhance tumor growth delay.

We performed a second *in vivo* study using a lower radiation dose in order to assess the time for tumors to grow from 7 mm to 12 mm, AGD. In this case, we increased the dose of sunitinib to 1.3 mg/mouse for 5 days and decreased the radiation dose to 1 Gy per fraction for 5 days (Figure 6B). All treatments prolonged the time for PC3 tumors to grow to 12 mm when compared to untreated controls (AGD): sunitinib alone delayed tumor growth by 19.1 (±5.3) days and radiation alone by 19.4 (± 9.0) days. Administration of sunitinib 1 h before each dose of radiation did not augment radiation induced tumor growth delay (AGD 20.7 ± 5.6 days); however sunitinib treatment initiated 24 h following the last dose of radiation did provide additional growth delay (AGD 38.0 ± 8.3 days) but this increase in AGD did not reach statistical significance when compared to radiation alone (*p* = 0.15). However, this second study confirmed the initial finding that the sequential treatment schedule with sunitinib administration following the completion of radiation treatment resulted in superior anti-tumor efficacy.

## Discussion

Previous reports have shown that interruption of VEGFR or PDGFR signaling can enhance the damaging effects of ionizing radiation [3]. For example, targeted therapy using cediranib, a small molecule VEGFR-inhibitor used in junction with radiotherapy, synergistically enhanced the growth delay of calu-6 lung xenografts and was associated with increased levels of apoptosis and necrosis in histological samples [13]. Cuneo et al. [10] demonstrated the effectiveness of combining sunitinib with radiation for the treatment of human pancreatic adenocarcinomas. Their results revealed that sunitinib or radiation when used alone delayed tumor growth, however when combined, the delay was significantly enhanced. Similar findings were reported for Lewis carcinomas treated *in vivo* with the combination of sunitinib and radiation [11]. Thus with prior reports illustrating the effectiveness of the combination of sunitinib and radiation on both cell lines and xenograft tumors, derived from a variety of human cancers, we investigated whether it would radiosensitize three prostate cell lines; the hormone independent DU145 and PC3 and hormone dependent androgen receptor expressing LNCaPs. This was of interest because the radioresistance of prostate cancer cells potentially limits the outcome of radiotherapy for this disease and inhibitors directed at the mechanisms of resistance might be of benefit.

Western blot analysis (Figure 1) showed that DU145 and PC3 cells express one or more of sunitinib’s cellular targets, i.e. VEGFR2, PDGFR- and c-Kit. Based on this, we postulated that sunitinib would radiosensitize these two cell lines but perhaps not radiosensitize the LNCaP cell line, found to express none of the given targets. This indeed turned out to be the case when sunitinib radiosensitization was assessed by clonogenic assay (Figure 3); DU145 and PC3 cells were modestly radiosensitized and LnCaP cells were not. However, in spite of the modest radiosensitization seen using sunitinib on DU145 and PC3 cells, the reduction in SF2 values observed would be predicted to have clinical impact in a fractioned treatment protocol in prostate cancer patients.

In spite of growing interest in combining novel tyrosine kinase inhibitors (TKIs) with conventional techniques such as radiotherapy, the molecular mechanisms by which TKIs elicit their sensitizing effects remain to be elucidated [14]. However, generally, it appears that many if not most TKIs inhibit signaling downstream of growth factor receptors mediated by the PI3K-AKT and Ras-Raf-MEK-ERK pathways [15,16]. Activation of both the ERK and AKT pathways are a frequent event in prostate cancers and a strong association between the expression of these kinases and poor prognosis is often observed [17,18]. Thus, we tested whether sunitinib suppressed p-AKT and/or p-ERK, 2 appropriate downstream elements of the signaling pathways under investigation. The results showed that sunitinib suppressed p-ERK in un-irradiated and irradiated DU145 and PC3 cells suggesting that radioresistance in these cells lines is mediated through the Ras-Raf-MEK-ERK pathway. This is consistent with numerous reports in the literature illustrating the importance of this pathway in governing radiation response in tumor cells [19].

Perhaps the most important mechanism for dictating the cytotoxicity of ionizing radiation involves the repair of radiation-induced DNA double strand breaks (DSBs) [20]. Repair of these lesions critically determines the degree of cell killing by radiation. Induction and repair of radiation-induced DSBs is commonly followed using the detection of γH2AX foci [21]. This assay is very sensitive and we have used it previously to demonstrate that the radiosensitizing action of other molecularly-targeted agents involves an inhibition of DSB repair detected on the basis of a prolongation of γH2AX foci in the agent plus radiation samples compared to radiation alone controls [12]. In the present study, however, we were unable to detect any prolongation of γH2AX foci by sunitinib (Figure 5) suggesting that sunitinib does not interfere with the repair of radiation-induced DSBs. This may not be too surprising since the degree of radiosensitization produced by sunitinib here is small compared to what was observed in our previous studies using other molecularly targeted agents. Thus, it is conceivable that sunitinib suppresses DSB repair to a small degree that is undetectable by this assay or that sunitinib radiosensitizes by some other mechanism.

Based on the experiments conducted *in vitro*, we hypothesized that daily sunitinib treatments concurrent with daily fractionated radiation would enhance tumor growth delay compared to radiation alone. However, sunitinib given concurrently with radiation did not prolong tumor growth delay. Conversely, when animals were treated with sunitinib commencing the day after fractionated radiation was complete, tumor growth delay was enhanced. We conclude that, at least in this treatment protocol and tumor model, sunitinib and radiation do not interact directly to radiosensitize the PC3 tumor cells *in vivo* as they did *in vitro* or that the modest degree of radiosensitization seen *in vitro* cannot be observed in the *in vivo* model. Alternatively, the anti-angiogenic activity of sunitinib may increase tumor hypoxia when administered prior to radiation thereby decreasing radiosensitivity and offsetting any radiosensitizing effect of the drug [22]. This possibility is supported by previous reports showing that sunitinib and other anti-angiogenic agents may enhance tumor blood vessel distruction during fractionated irradiation [11,23].

The fact that tumor growth delay was enhanced when sunitinib was given after radiotherapy was completed suggests that sunitinib may be acting on the irradiated tumor stroma and suppressing its ability to sustain regrowth of the irradiated tumor. This latter effect is consistent with previous reports illustrating enhanced tumor control when anti-angiogenic agents are applied after the completion of radiotherapy [11]. For example, Zips et al. reported that the adjuvant application of PTK787/ZK222584 preferentially retarded tumor growth when combined with fractionated irradiation [24-26]. Similar findings have been reported for other anti-angiogenic agents including bevacizumab, ZD6474 and sunitinib [11,23,27].

Our results demonstrate the effectiveness of sunitinib when combined with radiation for enhancing the radiosensitivity of androgen independent prostate cancer cells when treated *in vitro*. Although a mechanism mediating this response was not isolated, further studies into signaling functions downstream of sunitinib’s targeted growth factor receptors may ultimately provide greater insights. In the *in vivo* study, enhancement of tumor growth delay was not observed when sunitinib was given concurrently with fractionated radiation. However, tunor growth delay was enhanced when sunitinib treatment was initiated after the completion of fractionated radiation suggesting that sunitinib suppresses the ability of the tumor stroma to sustain regrowth of the irradiated tumor. Castrate-resistant clones can be a dilemma for radiation since the best outcomes depend on combination therapy with androgen deprivation to decrease tumor bulk locally and prevent or delay metastasis. The data submitted here and other reports in the literature suggest that the combination of TKIs such as sunitinib with radiation offers a promising approach. However, the effectiveness of such combinations may critically depend on appropriate scheduling of the agents.

## Conclusion

Sunitinib at doses of 100 nM and 250 nM modestly enhanced the radiosensitivity of DU145 and PC3 hormone-independent, human prostate cancer cell lines, respectively but did not sensitize the androgen-dependent cell line LNCaP. Sunitinib does not appear to mediate its radio-sensitizing effect via interruption of DNA repair. The fact that tumor growth delay was only enhanced when sunitinib was given after radiotherapy was completed suggests that sunitinib may be acting on the irradiated tumor stroma and suppressing its ability to sustain regrowth of the irradiated tumor rather than by radiosensitizing during radiation. Thus, based on the *in vivo* results, we believe that the combination of sunitinib and radiation offers a promising approach for treating human prostate cancer.

## Competing interests

The authors declare that they have no competing interests.

## Authors’ contributions

CB performed the *in vitro* experiments and drafted the manuscript. TS and KM conducted the *in vivo* experiments. KB performed statistical analysis and helped prepare the final manuscript. PW, DK and RM conceived the study, analyzed data, and prepared the final manuscript. All authors read and approved the final manuscript.
